# A Contribution to Knowledge of *Craterellus* (Hydnaceae, Cantharellales) in China: Three New Taxa and Amended Descriptions of Two Previous Species

**DOI:** 10.3389/fmicb.2022.906296

**Published:** 2022-07-13

**Authors:** Yu-Zhuo Zhang, Ping Zhang, Bart Buyck, Li-Ping Tang, Zhi-Qun Liang, Ming-Sheng Su, Yan-Jia Hao, Hong-Yan Huang, Wen-Hao Zhang, Zuo-Hong Chen, Nian-Kai Zeng

**Affiliations:** ^1^College of Science, Hainan University, Haikou, China; ^2^Key Laboratory of Tropical Translational Medicine of Ministry of Education, School of Pharmacy, Hainan Medical University, Haikou, China; ^3^College of Life Science, Hunan Normal University, Changsha, China; ^4^Institut Systématique, Evolution, Biodiversité (ISYEB), UMR 7205, Muséum National d’ Histoire Naturelle, CNRS, Sorbonne Université, Paris, France; ^5^School of Pharmaceutical Sciences and Yunnan Key Laboratory of Pharmacology for Natural Products, Kunming Medical University, Kunming, China; ^6^Key Laboratory of Prevention and Treatment of Cardiovascular and Cerebrovascular Diseases of Ministry of Education, Gannan Medical University, Ganzhou, China; ^7^School of Horticulture, Anhui Agricultural University, Hefei, China

**Keywords:** East Asia, molecular phylogeny, morphology, new taxa, taxonomy

## Abstract

Species of *Craterellus* (Hydnaceae, Cantharellales) in China are investigated on the basis of morphological and molecular phylogenetic analyses of DNA sequences from nuc 28S rDNA D1-D2 domains (28S) and nuc rDNA internal transcribed spacer ITS1-5.8S-ITS2 region. Five species are recognized in China, of which three of them are described as new, viz. *C. fulviceps*, *C. minor*, and *C. parvopullus*, while two of them are previously described taxa, viz. *C. aureus*, and *C. lutescens*. A key to the known Chinese taxa of the genus is also provided.

## Introduction

*Craterellus* Pers. (Hydnaceae, Cantharellales), typified by *C. cornucopioides* (L.) Pers., is characterized by a small, funnel-shaped basidioma with a hollow stipe (Petersen, 1979a). Recent molecular phylogenetic data have confirmed the monophyly of the genus (Hibbett et al., 2014). To date, many taxa of *Craterellus* have been discovered in Africa, America, and Asia (Dahlman et al., 2000; Matheny et al., 2010; Beluhan and Ranogajec, 2011; Kumari et al., 2012; Wilson et al., 2012; Das et al., 2017; Hembrom et al., 2017; Bijeesh et al., 2018; Zhong et al., 2018; Zhang et al., 2020; Cao et al., 2021a,b). They have received much attention for their edibility and medicinal value; for example, *C. cornucopioides* is considered a highly nutritious edible fungus and has antihyperglycemic, antioxidative, and antitumor activities (Beluhan and Ranogajec, 2011; Liu et al., 2012; Fan et al., 2014), and *C. tubaeformis* (Fr.) Quél. has antioxidant, antimicrobial, and anti-inflammatory activities (Li, 1996; O’Callaghan et al., 2014).

**FIGURE 1:**
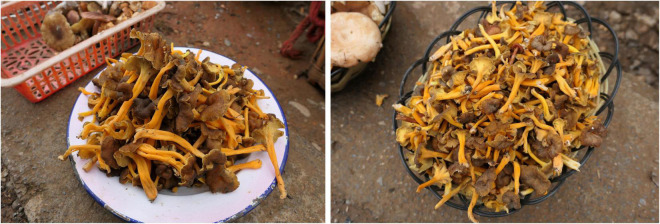
Collections of *Craterellus lutescens* sold as edibles in the market of Yunnan Province, southwestern China. Photos: H.-Y. Huang.

A total of thirteen taxa of *Craterellus* have been described/reported from China in previous studies, viz. *C. albidus* Chun Y. Deng, M. Zhang & Jing Zhang, *C. atrobrunneolus* T. Cao & H.S. Yuan, *C. aureus* Berk. & M.A. Curtis., *C. badiogriseus* T. Cao & H.S. Yuan, *C. croceialbus* T. Cao & H.S. Yuan, *C. cornucopioides*, *C. cornucopioides* var. *parvisporus* Heinem., *C. lutescens* (Fr.) Fr., *C. luteus* T.H. Li & X.R. Zhong, *C. odoratus* (Schwein.) Fr., *C. macrosporus* T. Cao & H.S. Yuan, *C. squamatus* T. Cao & H.S. Yuan, and *C. tubaeformis* (Li, 1996, 2005; Beluhan and Ranogajec, 2011; Xiao et al., 2012; Zhang et al., 2020; Cao et al., 2021a,b). Most of them are well known in the country, for mushrooms identified as *C. aureus*, *C. cornucopioides*, *C. cornucopioides* var. *parvisporus*, *C. lutescens*, or *C. tubaeformis* are sold as edibles in the market of Yunnan Province, southwestern China (Wang et al., 2004; Zhang et al., 2021; Figure 1). In addition, interesting compounds such as merosesquiterpenids, acetylenic acids, and derivatives have been isolated from collections identified as *C. lutescens* and *C. odoratus* in the country (Zhang et al., 2010; Huang et al., 2016, 2017).

Recently, lots of collections of *Craterellus* in China have been made, which were studied using morphological and molecular phylogenetic analyses. The aim was to (i) describe new taxa and (ii) reevaluate some reports of previously described taxa.

## Materials and Methods

### Morphological Studies

Field notes and digital photographs were made from fresh specimens which were dried and deposited in the Fungal Herbarium of Hainan Medical University (FHMU) (Index Herbariorum), Haikou City, Hainan Province of China. Color codes follow Kornerup and Wanscher (1981). An optical light microscope (CX23, Olympus, Tokyo, Japan) was used to observe and measure the microstructures of basidiomata; the samples were hand-sectioned and mounted in a 5% KOH solution. The notation [n/m/p] indicates “n” basidiospores measured from “m” basidiomata of “p” collections. Dimensions of basidiospores are presented as (a–)b–e–c(–d), where the range “b–c” represents a minimum of 90% of the measured values (5th to 95th percentile), and extreme values (a and d), whenever present (a <5th percentile, d >95th percentile), are in parentheses, “e” refers to the average length/width of basidiospores. “Q” refers to the length/width ratio of basidiospores; “Q_m_” refers to the average “Q” of basidiospores and is presented with standard deviation. The terms referring to the size of basidioma are based on Bas (1969).

### Molecular Procedures

Total genomic DNA was extracted from dried basidiomata (10–20 mg) using the Plant Genomic DNA Kit (CWBIO, Beijing, China) according to the manufacturer’s instructions. Protocols for polymerase chain reaction (PCR) amplification and sequencing followed An et al. (2017). The universal primer pairs ITS5/ITS4 (White et al., 1990) and LR0R/LR5 (Vilgalys and Hester, 1990; James et al., 2006) were used for PCR amplification of nuclear ribosomal internal transcribed spacer (ITS) and large subunit ribosomal DNA (28S), respectively. PCR conditions followed Zhang et al. (2021). PCR products were checked using 1% (w/v) agarose gel electrophoresis. The amplified PCR products were sequenced using an ABI 3730 DNA Analyzer (BGI, Guangzhou, China) with the PCR primers. Forward or reverse sequences were assembled with BioEdit (Hall, 1999). All newly obtained sequences were deposited in GenBank^1^.

### Dataset Assembly

A total of thirty DNA sequences (16 of 28S, 14 of ITS) from 17 collections were newly generated for this study (Table 1). For the concatenated dataset, the 28S and ITS sequences generated in the study were aligned with selected sequences from previous studies and GenBank (Table 1). *Hydnum minus* FHMU2461 and *Hydnum cremeoalbum* FHMU2153 were chosen as outgroups as described by An et al. (2017). Sequences of 28S and ITS were aligned separately to test for phylogenetic conflict. The topologies of the phylogenetic trees based on a single gene were identical, indicating that the phylogenetic signals present in the different gene fragments were not in conflict. Then, the sequences of the different genes were aligned using MUSCLE (Edgar, 2004), and alignments were purged from unreliably aligned positions and gaps using Gblocks (Castresana, 2000). The sequences of the different genes were concatenated using Phyutility v2.2 for further analyses (Smith and Dunn, 2008).

**TABLE 1:** List of collections used in this study.

Taxon	Voucher	Locality	GenBank accession no.		References
			28S	ITS	
*Craterellus* aff. *excelsius*	G3184	Guyana	KJ786602	—	Unpublished
*C.* aff. *excelsius*	G3279	Guyana	KJ786625	—	Unpublished
*C.* aff. *tubaeformis*	Mushroom Observer # 289652	Mexico	—	MH168540	Unpublished
*C. albidus*	HGASMF01-3581	Guizhou, SW China	MT921161	—	Zhang et al., 2020
*C. albidus*	HGASMF01-10046	Guizhou, SW China	MT921162	—	Zhang et al., 2020
*C. albostrigosus*	CAL 1624	India	MG593194	—	Bijeesh et al., 2018
*C. atratoides*	TH8243	Guyana	—	KT339209	Wilson et al., 2012
*C. atratoides*	MCA1313	Guyana	JQ915119	JQ915093	Wilson et al., 2012
*C. atratoides*	TH9232	Guyana	JQ915137	JQ915111	Wilson et al., 2012
*C. atratoides*	TH8473	Guyana	JQ915129	JQ915103	Wilson et al., 2012
*C. atratoides*	AMV1965a	Colombia	KT724157	KT724106	Unpublished
*C. atratoides*	AMV1959	Colombia	KT724156	—	Unpublished
*C. atratoides*	AMV1870	Colombia	—	KT354698	Unpublished
*C. atratoides*	AMV1992	Colombia	—	KT354700	Unpublished
*C. atratoides*	AMV1990	Colombia	—	KT354699	Unpublished
*C. atratus*	AMV1832	Colombia	KT724158	KT724107	Unpublished
*C. atratus*	TH9203	Guyana	JQ915133	JQ915107	Wilson et al., 2012
*C. atratus*	MCA990	Guyana	JQ915126	JQ915100	Wilson et al., 2012
*C. atratus*	MCA1070	Guyana	JQ915118	JQ915092	Wilson et al., 2012
*C. atratus*	MN21-2006 (envir. seq.)	Thailand	—	AB445115	Disyatat et al., 2016
*C. atrobrunneolus*	Yuan13878	Yunnan, SW China	MN894058	MN902353	Cao et al., 2021a
*C. atrocinereus*	Arora15001	United States	—	KR560049	Frank, 2015
*C. atrocinereus*	JLF3750	United States	—	KR560048	Frank, 2015
***C. aureus***	**N.K. Zeng1057** **(FHMU2407)**	**Hainan, southern China**	**OL439672**	**OM469019**	**Present study**
***C. aureus***	**M.S. Su145** **(FHMU6549)**	**Jiangxi, eastern China**	**OL439673**	**—**	**Present study**
***C. aureus***	**N.K. Zeng3141** **(FHMU2102)**	**Hainan, southern China**	**OL439674**	**OM469020**	**Present study**
***C. aureus***	**N.K. Zeng3139** **(FHMU2100)**	**Hainan, southern China**	**OL439675**	**—**	**Present study**
***C. aureus***	**M.S. Su196** **(FHMU6550)**	**Jiangxi, eastern China**	**OL439676**	**OL439545**	**Present study**
*C. badiogriseus*	Yuan 14776	Liaoning, NE China	MW979532	MW980548	Cao et al., 2021b
*C. badiogriseus*	Yuan 14779	Liaoning, NE China	MW979533	MW980549	Cao et al., 2021b
*C. caeruleofuscus*	MH17001	United States	MT237468	MH558300	Cao et al., 2021a
*C. calicornucopioides*	JLF3744	United States	—	KR560046	Frank, 2015
*C. calicornucopioides*	Arora 15002	United States	—	KR560047	Frank, 2015
*C. calyculus*	Mushroom Observer # 321697	United States	—	MK607596	Unpublished
*C. carolinensis*	FLAS-F-59997	United States	—	KY654712	Petersen, 1969
*C.* cf. *lutescens*	BB 13.048	Canada	KM484696	—	Shao et al., 2014
*C.* cf. *tubaeformis*	BB 13.125	United States	KM484697	—	Shao et al., 2014
*C. cinereofimbriatus*	TH9264	Guyana	JQ915138	JQ915112	Wilson et al., 2012
*C. cinereofimbriatus*	TH9075	Guyana	JQ915131	JQ915105	Wilson et al., 2012
*C. cinereofimbriatus*	TH9264	Guyana	JQ915138	JQ915112	Wilson et al., 2012
*C. cinereofimbriatus*	TH8999	Guyana	JQ915130	JQ915104	Wilson et al., 2012
*C. cinereofimbriatus*	JOH4	Colombia	KT724159	—	Unpublished
*C. cinereus*	107-08	India	JF412276	JF412278	Kumari et al., 2012
*C. cinereus*	AST2015	Pakistan	—	MF374488	Naseer and Khalid, 2018
*C. cinereus*	AST12B	Pakistan	—	MF374489	Naseer and Khalid, 2018
*C. cornucopioides*	HbO-53302	Norway	AF105301	—	Dahlman et al., 2000
*C. cornucopioides*	UPSF-11792	Sweden	AF105297	—	Dahlman et al., 2000
*C. cornucopioides*	Groc11399 clone 1	United States	—	KT693262	Raja et al., 2017
*C. cornucopioides*	WA0000071019	Poland	—	MK028881	Kotowski et al., 2019
*C. cornucopioides*	AFTOL-ID 286	United States	AY700188	DQ205680	Matheny et al., 2007, 2010
*C. cornucopioides*	—	Tibet, SW China	AJ279572	—	Li et al., 1999
*C. cornucopioides*	CNF 1/7292	Croatia	—	MK169230	Mešić et al., 2020
*C. croceialbus*	Yuan 14623	Liaoning, NE China	MW979529	MW980572	Cao et al., 2021b
*C. croceialbus*	Yuan 14647	Liaoning, NE China	MW979530	MW980573	Cao et al., 2021b
*C. cornucopioides* var. *mediosporus*	268-06	India	JF412275	JF412277	Kumari et al., 2012
*C. excelsus*	TH8235	Guyana	JQ915128	JQ915102	Wilson et al., 2012
*C. excelsus*	TH7515	Guyana	JQ915127	JQ915101	Wilson et al., 2012
*C. excelsus*	MCA3107	Guyana	JQ915121	JQ915095	Wilson et al., 2012
*C. fallax*	PBM3290	United States	—	GU590923	Matheny et al., 2010
*C. fallax*	MQ15002	Canada	—	MH571125	Unpublished
*C. fallax*	FLAS-F-60401	United States	—	MH281835	Unpublished
***C. fulviceps***	**MHHNU10567** **(FHMU6553)**	**Hunan, central China**	**OL439678**	**OL439548**	**Present study**
*C. ignicolor*	UPSF-11794	United States	AF105314	—	Dahlman et al., 2000
*C. indicus*	PUN3884	India	HM113529	HM113530	Kumari et al., 2012
*C. indicus*	MSR6	India	—	HQ450769	Kumari et al., 2012
*C. inusitatus*	CAL 1625	India	MG593195	—	Bijeesh et al., 2018
*C. lutescens*	104198 (envir. seq.)	Ireland	—	AY082606	Harrington and Mitchell, 2002
*C. lutescens*	TM02_22	Canada	EU522746	—	Porter et al., 2008
*C. lutescens*	UPSF-11790	Sweden	AF105303	—	Dahlman et al., 2000
*C. lutescens*	UPSF-11791	Spain	AF105304	—	Dahlman et al., 2000
*C. lutescens*	SS575	Sweden	JQ976982	—	Tibuhwa et al., 2012
*C. lutescens*	ma023	Italy	MN592820	MN595294	Federico et al., 2020
***C. lutescens***	**L.P. Tang1647** **(FHMU6547)**	**Yunnan, SW China**	**OL439679**	**OL439549**	**Present study**
***C. lutescens***	**L.P. Tang1705** **(FHMU6548)**	**Yunnan, SW China**	**OL439680**	**—**	**Present study**
***C. lutescens***	**W.H. Zhang441-1** **(FHMU6544)**	**Yunnan, SW China**	**OL439681**	**OL439550**	**Present study**
***C. lutescens***	**W.H. Zhang441-2** **(FHMU6545)**	**Yunnan, SW China**	**OL439682**	**OL439551**	**Present study**
***C. lutescens***	**W.H. Zhang441-3** **(FHMU6546)**	**Yunnan, SW China**	**OL439683**	**OL439552**	**Present study**
*C. luteus*	GDGM46432	Guangdong, southern China	MG727898	MG727897	Zhong et al., 2018
*C. luteus*	GDGM48105	Guangdong, southern China	MG701171	MG727896	Zhong et al., 2018
*C. luteus*	GDGM49495	Guangdong, southern China	MG806926	MG806930	Zhong et al., 2018
*Craterellu macrosporus*	Yuan 14782	Liaoning, NE China	MW979531	MW980574	Cao et al., 2021b
*C. melanoxeros*	SS576	Sweden	JQ976983	—	Tibuhwa et al., 2012
“*C. melanoxeros*”	420526MF0891	China	MG712381	—	Unpublished
***C. minor***	**MHHNU32505** **(FHMU6554)**	**Hunan, central China**	**OL439684**	**OL439553**	**Present study**
*C. odoratus*	14026h2	United States	MN227279	—	Unpublished
*C. odoratus*	14026h1	United States	MN227278	—	Unpublished
*C. odoratus*	UPSF-11799	United States	AF105306	—	Dahlman et al., 2000
*C. olivaceoluteus*	TH9205	Guyana	JQ915135	JQ915109	Wilson et al., 2012
*C. olivaceoluteus*	MCA3186	Guyana	JQ915124	JQ915098	Wilson et al., 2012
*C. parvogriseus*	CAL1533	India	MF421098	MF421099	Das et al., 2017
*C. parvogriseus*	KNPS_WC18158	Korea	MT974136	—	Ko et al., 2020
***C. parvopullus***	**N.K. Zeng4913** **(FHMU6555)**	**Hainan, southern China**	**OL439685**	**OM334829**	**Present study**
***C. parvopullus***	**N.K. Zeng4912** **(FHMU6556)**	**Hainan, southern China**	**OL439686**	**OM334828**	**Present study**
***C. parvopullus***	**N.K. Zeng4911** **(FHMU6557)**	**Hainan, southern China**	**OL439687**	**OM334827**	**Present study**
*C. pleurotoides*	MCA3124	Guyana	JQ915123	JQ915097	Wilson et al., 2012
*C. pleurotoides*	TH9220	Guyana	JQ915136	JQ915110	Wilson et al., 2012
*C. shoreae*	CAL_F_1396	India	KY290585	—	Cao et al., 2021a
*C. sinuosus*	TF1802	United States	U87992	—	Feibelman et al., 1997
***Craterellus* sp.**	**Y.J. Hao2080** **(FHMU6551)**	**Anhui, eastern China**	**—**	**OL439546**	**Present study**
***Craterellus* sp.**	**MHHNU32154** **(FHMU6552)**	**Anhui, eastern China**	**OL439677**	**OL439547**	**Present study**
*Craterellus* sp.	RSEM26_17 (envir. seq.)	Austria	EU046070	—	Urban et al., 2008
*Craterellus* sp.	RSEM16_35 (envir. seq.)	Austria	EU046065	—	Urban et al., 2008
*Craterellus* sp.	RSEM15_01 (envir. seq.)	Austria	EU046056	—	Urban et al., 2008
*Craterellus* sp.	RSEM26 (envir.seq.)	Austria	EU046028	—	Urban et al., 2008
*Craterellus* sp.	RSEM26_64 (envir. seq.)	Austria	EU046073	—	Urban et al., 2008
*Craterellus* sp.	RSEM26_17 (envir.seq.)	Austria	EU046070	—	Urban et al., 2008
*Craterellus* sp.	AWW263	Malaysia	JQ915117	JQ915091	Wilson et al., 2012
*Craterellus* sp.	610723MF0035	—	—	KY950471	Unpublished
*Craterellus* sp.	LAM 0257	Malaysia	KY091022	—	Unpublished
*Craterellus* sp.	LAM 0254	Malaysia	KY091020	—	Unpublished
*Craterellus* sp.	DOB 2489	Malaysia	KY090820	—	Unpublished
*Craterellus* sp.	NC-8338	United States	—	AY456340	Edwards et al., 2004
*Craterellus* sp.	CY14_025_1 (envir. seq.)	New Caledonia	—	KY774189	Carriconde et al., 2019
*Craterellus* sp.	PGK14_052 (envir. seq.)	New Caledonia	—	KY774191	Carriconde et al., 2019
*Craterellus* sp.	16450	India	—	MF589901	Unpublished
*Craterellus* sp.	Mushroom Observer # 289663	Mexico	MH223620	—	Unpublished
*Craterellus* sp.	YM226 (envir.seq.)	Japan	—	AB848480	Miyamoto et al., 2014
*Craterellus* sp.	CM13_278_1 (envir. seq.)	New Caledonia	—	KY774188	Carriconde et al., 2019
*Craterellus* sp.	OTU_506s (envir. seq.)	Europe	—	MT095625	Arraiano-Castilho et al., 2020
*Craterellus* sp.	CYMy31E2 (envir. seq.)	New Caledonia	—	KY774190	Carriconde et al., 2019
*Craterellus* sp.	G3154	Guyana	KJ786597	KJ786692	Unpublished
*Craterellus* sp.	G2070	Guyana	—	KJ786682	Unpublished
*Craterellus* sp.	G3228	Guyana	KJ786613	—	Unpublished
*Craterellus* sp.	G3237	Guyana	KJ786614	KJ786704	Unpublished
*Craterellus* sp.	G3112	Guyana	KJ786587	—	Unpublished
*Craterellus* sp.	G1340	Guyana	KJ786565	KJ786670	Unpublished
*Craterellus* sp.	BB 09.079	New Caledonia	KM484695	—	Shao et al., 2014
*Craterellus* sp.	LM3266	France	—	KM576330	Shao et al., 2014
*Craterellus* sp.	AMV1879	Colombia	KT724161	—	Unpublished
*Craterellus* sp.	M66A9 (envir. seq.)	Mexico	—	EU563479	Morris et al., 2008
*Craterellus* sp.	LMAC6b-09	France	—	JF506753	Unpublished
*Craterellus* sp.	YM835	Japan	—	LC175080	Miyamoto et al., 2018
*Craterellus* sp.	14044	Spain	—	MW282673	Unpublished
*Craterellus* sp.	OTU_236	Germany	—	MW238032	Unpublished
*Craterellus* sp.	MEL:2382717	Australia	—	KP012898	Unpublished
*Craterellus* sp.	MEL:2383015	Australia	—	KP012867	Unpublished
*Craterellus* sp.	ECM90 (envir. seq.)	Zhejiang, eastern China	—	JQ991715	Unpublished
*C. squamatus*	Yuan 14520	Liaoning, NE China	MW979534	MW980571	Cao et al., 2021b
*C. squamatus*	Yuan 14721	Liaoning, NE China	MW979535	MW980570	Cao et al., 2021b
*C. strigosus*	TH9204	Guyana	JQ915134	JQ915108	Wilson et al., 2012
*C. strigosus*	MCA1750	Guyana	JQ915120	JQ915094	Wilson et al., 2012
*C. strigosus*	JOH16 (envir. seq.)	Colombia	—	KT354701	Unpublished
*C. strigosus*	AMV1885 (envir. seq.)	Colombia	KT724164	KT724110	Unpublished
*C. tubaeformis*	DAVFP26257	Canada	—	HM468491	Zhou et al., 2011
*C. tubaeformis*	MushroomObserver.org/230696	United States	—	MH298913	Unpublished
*C. tubaeformis*	MushroomObserver.org/312399	United States	—	MH063270	Unpublished
*C. tubaeformis*	2A4	Japan	AB973798	AB973799	Unpublished
*C. tubaeformis*	1D3	Japan	—	AB973729	Unpublished
*C. tubaeformis*	UPS-11797	United States	AF105311	—	Dahlman et al., 2000
*C. tubaeformis*	TRTC52516	Belgium	—	HM468496	Zhou et al., 2011
*C. tubaeformis*	DM1094	Denmark	—	MT640258	Unpublished
*C. tubaeformis*	UPSF-11793	Sweden	AF105307	—	Dahlman et al., 2000
*C. tubaeformis*	BB 07.293	Slovakia	KF294640	—	Buyck et al., 2014
*C. tubaeformis*	TRTC52235	Belgium	—	HM468497	Zhou et al., 2011
*C. tubaeformis*	BR089347	Canada	—	HM468493	Zhou et al., 2011
*C. tubaeformis*	OSC-41280	United States	AF105313	—	Dahlman et al., 2000
*C. tubaeformis*	GCB1905	Belgium	—	MT004784	Dahlman et al., 2000
*C. tubaeformis*	UPSF-11795	United States	AF105308	—	Dahlman et al., 2000
*Hydnum* sp.	N.K. Zeng2819 (FHMU2461)	Yunnan, SW China	KY407528	KY407533	An et al., 2017
*Hydnum* sp.	N.K. Zeng2511 (FHMU2153)	Hainan, southern China	KY407527	KY407532	An et al., 2017

*GenBank numbers in bold indicate the newly generated sequences; SW, Southwest; NE, Northeast.*

### Phylogenetic Analyses

The combined nuclear dataset (28S + ITS) was analyzed using maximum likelihood (ML) and Bayesian inference (BI) methods. ML tree generation and bootstrap (BS) analyses were performed using RAxML v7.2.6 (Stamatakis, 2006), running 1,000 replicates combined with the ML search. BI was conducted in MrBayes v3.1 (Huelsenbeck and Ronquist, 2005) on the CIPRES Science Gateway portal (Miller et al., 2011). The best-fit likelihood models of 28S (GTR + I + G) and ITS (HKY + I + G) were estimated in MrModeltest v2.3 (Nylander, 2004) based on the Akaike information criterion. Bayesian analysis was repeated for 30 million generations and sampled every 1,000 generations. Trees sampled from the first 25% generations were discarded as burn-in, and Bayesian posterior probabilities (PP) were then calculated for a majority-rule consensus tree of the retained sampled trees.

## Results

### Molecular Data

**FIGURE 2:**
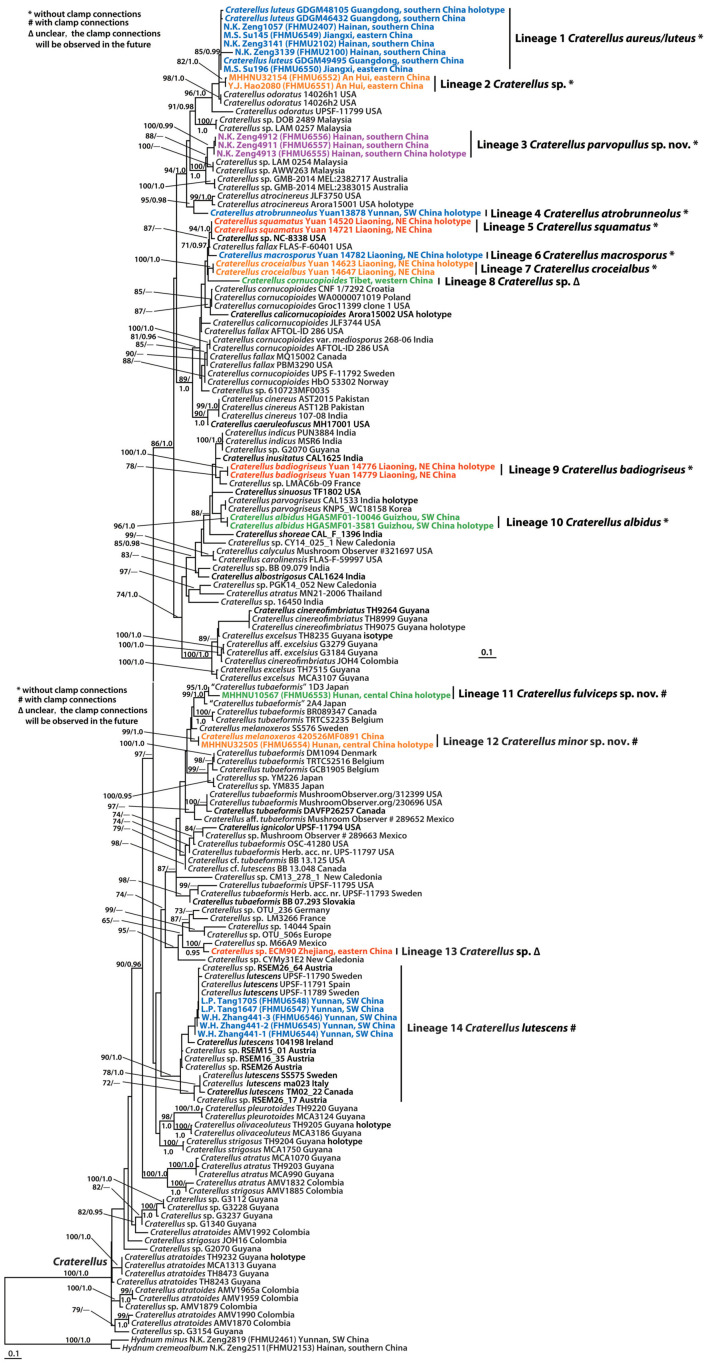
Phylogram inferred from a combined dataset (28S + ITS) of *Craterellus* using RAxML. RAxML bootstrap percentages (BS ≥ 70%) and Bayesian posterior probabilities (PP ≥ 0.95) are indicated above or below the branches as BS/PP.

The combined dataset (28S + ITS) of *Craterellus* consisted of 161 taxa and 2,173 nucleotide sites (Figure 2), and the alignment was submitted to TreeBase (S28981). The topologies of the phylogenetic trees based on the combined dataset generated from ML and BI analyses were identical, but statistical support showed slight differences. In this study, we focused on lineages 1–14 from China (Figure 2). Lineage 1, with strong statistical support (BS = 85%, PP = 0.99), comprised of three collections (GDGM46432, GDGM48105, and GDGM49495) of *C. luteus*, and three collections (FHMU2100, FHMU2102, and FHMU2407) from southern China, and two collections (FHMU6549, FHMU6550) from eastern China. Lineage 2, with strong statistical support (BS = 82%, PP = 1.0), comprised of two collections (FHMU6551, and FHMU6552) from eastern China. Lineage 3, three collections (FHMU6555, FHMU6556, and FHMU6557) from southern China grouped together with high statistical support (BS = 100%, PP = 0.99). Lineage 4 comprised of the holotype of *C. atrobrunneolus.* Lineage 5, with strong statistical support (BS = 94%, PP = 1.0), comprised of two collections (Yuan 14,520, and Yuan 14,721) of *C. squamatus* from northeastern China. Lineage 6 comprised of the holotype of *C. macrosporus.* Lineage 7, with strong statistical support (BS = 100%, PP = 1.0), comprised of two collections (Yuan 14,623, and Yuan 14,647) of *C. croceialbus* from northeastern China. Lineage 8 comprised of one collection named *C. cornucopioides* from western China. Lineage 9, with strong statistical support (BS = 100%, PP = 1.0), comprised of two collections (Yuan 14,776 and Yuan 14,779) of *C. badiogriseus* from northeastern China. Lineage 10, with strong statistical support (BS = 96%, PP = 1.0), comprised of two collections (HGASMF01-10046, and HGASMF01-3581) of *C. albidus* from southwestern China. Lineage 11, with strong statistical support (BS = 95%, PP = 1.0), comprised of one collection (FHMU6553) from central China, and one collection labeled as *C. tubaeformis* from Japan. Lineage 12, with strong statistical support (BS = 99%, PP = 1.0), comprised of one collection (FHMU6554) from central China, and one collection labeled as *C. melanoxeros* also from China. Lineage 13 comprised of one collection (ECM90) from eastern China. Lineage 14, with strong statistical support (BS = 90%, PP = 1.0), comprised of seven collections of *C. lutescens* (UPSF-11789, UPSF-11790, UPSF-11791, 104198, SS575, ma023, and TM02_22), five collections labeled as *Craterellus* sp. (RSEM15_01, RSEM16_35, RSEM26, RSEM26_17, and RSEM26_64), and five collections (FHMU6544–FHMU6548) from southwestern China.

### Taxonomy

***Craterellus aureus*** Berk. & M.A. Curtis, Proc. Amer. Acad. Arts & Sci. 4: 123, 1860 Figures 3A–E, 4.

**FIGURE 3:**
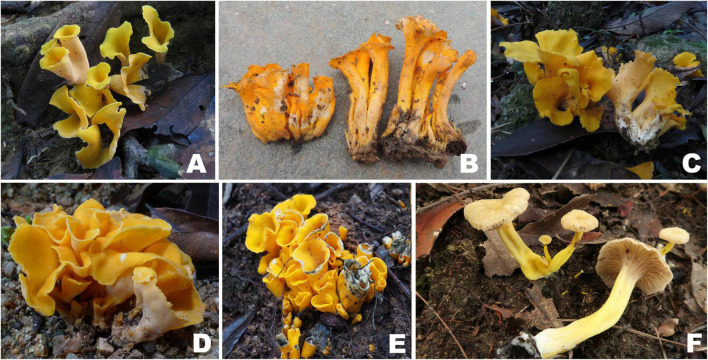
Basidiomata of *Craterellus* species. **(A–E)** *C. aureus* **(A)** FHMU2100; **(B)** FHMU6549; **(C)** FHMU2102; **(D)** FHMU2407; **(E)** FHMU6550; **(F)** *C. fulviceps* (FHMU6553, holotype). Photos: **(A,C,D)** N.-K. Zeng; **(B,E)** M.-S. Su; **(F)** P. Zhang.

**FIGURE 4:**
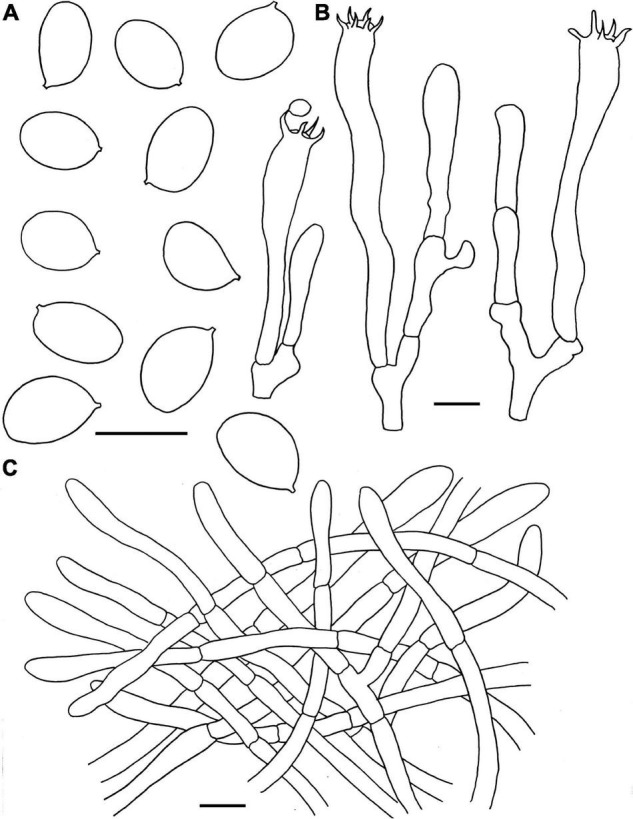
Microscopic features of *Craterellus aureus* (FHMU2407). **(A)** Basidiospores. **(B)** Basidia. **(C)** Pileipellis. Scale bars = 10 μm. Drawings by Y.-Z. Zhang.

**Basidiomata** medium-sized. **Pileus** 1.5–5 cm diam, infundibuliform, broadly infundibuliform with age; surface dry, vivid yellow (1A5) to orange (3A7); margin straight when young, wavy or lobed at maturity. **Hymenophore** nearly smooth, dirty white (1B2), yellow (4A7) to pale orange (1A2); context 0.1–0.15 cm in thickness, whitish (3A1) to pale yellow (3A2). **Stipe** 1.2–2.7 × 0.35–0.45 cm, central, hollow, usually curved, without any obvious demarcation between pileus and stipe; surface dry, yellowish-white (3A2), yellow (4A4) to pale orange (1A2). Basal mycelium white. **Odor** mild. **Spore print** not obtained.

**Basidiospores** [60/9/5] (7–)7.5–8.21–9(–9.5) × 5.5–5.97–6.5(–7) μm, Q = (1.17–)1.23–1.55(–1.64), Q_m_ = 1.38 ± 0.1, ellipsoid to broadly ellipsoid, smooth, slightly thick-walled (up to 0.5 μm), hyaline or yellowish in KOH. **Basidia** 50–83 × 6.5–8.5 μm, cylindro-clavate, with irregular flexuous, slightly thick-walled (up to 0.5 μm), 4–6-spored, pale yellowish in KOH; sterigmata 5–6 μm in length. **Cystidia** absent. **Pileipellis** intricate trichoderm composed of cylindrical, 4–9 μm wide, slightly thick-walled (0.5–0.7 μm) hyphae, faintly pale yellow in KOH; terminal cells 27–59 × 4–8 μm, subcylindrical to subclavate with obtuse apex. **Clamp connections** absent in all tissues.

Habitat: Gregarious, caespitose, or rarely solitary on the ground of forests dominated by *Castanea* spp. and *Quercus* spp. (Zhong et al., 2018).

Known distribution: Eastern China (Jiangxi Province), and southern China (Guangdong and Hainan Provinces, Hong Kong) (Berkeley and Curtis, 1860).

Specimens examined: CHINA. Hainan Province: Jianfengling of Hainan Tropical Rainforest National Park, elev. 850 m, 4 July 2012, N.K. Zeng1057 (FHMU2407); Limushan of Hainan Tropical Rainforest National Park, elev. 750 m, 27 July 2017, N.K. Zeng3139, 3141 (FHMU2100, 2102). Jiangxi Province: Ganzhou City, Shangyou Town, Youshixiangmeiling Village, elev. 180 m, 9 June 2016, M.S. Su145 (FHMU6549); Nanchang City, Wanli District, Zhaoxian Town, Dongyuan Village, elev. 180 m, 24 June 2018, M.S. Su196 (FHMU6550).

Notes: Our recent collections and the holotype of *C. luteus*, a species originally described from Guangdong Province, southern China (Zhong et al., 2018), phylogenetically group together with high statistical support (Figure 2), which suggests that these new specimens belong to *C. luteus*. Morphologically, these newly collected materials easily remind us of *C. aureus*, a species first described in Hong Kong, southern China. When *C. luteus* was first described (Zhong et al., 2018), the species looked different from the original diagnosis of *C. aureus* (Berkeley and Curtis, 1860; Corner, 1966): the bright yellow cap, large size, and robust aspect of the basidiomata and the white hymenophore made it impossible to associate *C. luteus* with Berkeley and Curtis’ original description. Our new collections, which share near-identical (BS = 83%, PP = 1.0) sequences with the holotype of *C. luteus*, indicate that this species might be more variable in overall aspect and color, thereby, significantly reducing the morphological differences with the orange *C. aureus*. Our collections also have a near-identical basidiospore size compared with those reported for *C. aureus*, whereas basidiospores of *C. luteus* are longer [(8.5–)9–11(–12.5) μm]. The fact that both species were described from southern China, sharing the same climate and vegetation, suggests *C. luteus* is a synonym of *C. aureus*, but it does not exclude the presence of a larger species complex in southern China within this clade.

The phylogenetic analyses also showed that *C. aureus* is closely related to *C. odoratus* (Schwein.) Fr. (Figure 2), a species originally described in North America (Petersen, 1979b; Knopf, 1981). However, *C. odoratus* has a more fragile basidioma, narrower basidiospores measuring 8.9–11.8 × 4.4–6.3 μm, and a strong pleasant odor (Petersen, 1979b; Knopf, 1981).

**FIGURE 5:**
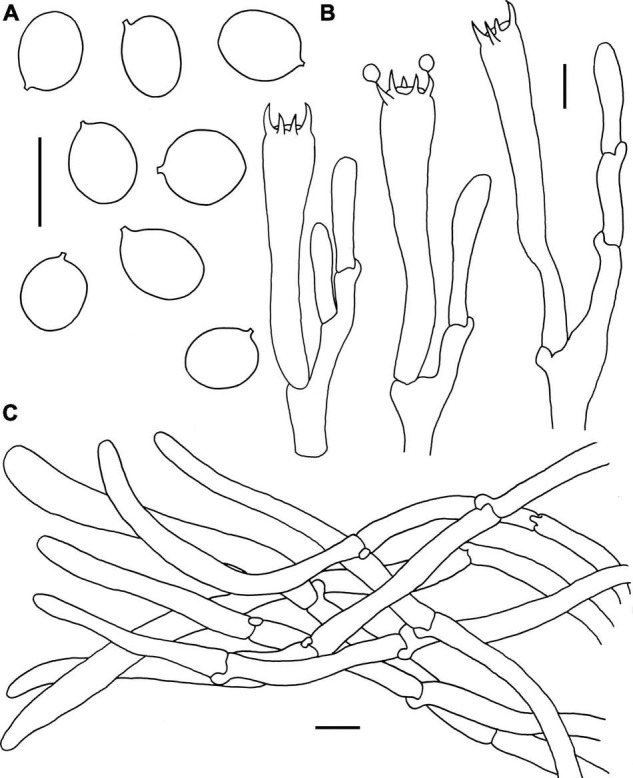
Microscopic features of *Craterellus fulviceps* (FHMU6553, holotype). **(A)** Basidiospores. **(B)** Basidia. **(C)** Pileipellis. Scale bars = 10 μm. Drawings by Y.-Z. Zhang.

***Craterellus fulviceps*** N.K. Zeng, Y.Z. Zhang, P. Zhang & Zhi Q. Liang, sp. nov. Figures 3F, 5 MycoBank: MB841969.

Diagnosis: This species is distinguished from others in *Craterellus* by its very small-sized basidioma, a fulvous pileus, a veined hymenophore, an egg-yolk yellow stipe, and a presence of clamp connections in all parts of the basidioma.

Etymology: Latin “*fulvi-*,” meaning fulvous, and “*ceps*,” meaning pileus, refer to the fulvous pileus of our new species.

Holotype: CHINA. Hunan Province: Rucheng County, Jiulongjiang Nature Reserve, elev. 600 m, 2 October 2020, P. Zhang MHHNU10567 (FHMU6553). GenBank accession number: 28S = OL439678, ITS = OL439548.

**Basidiomata** very small-sized. **Pileus** 1–3 cm diam, convex to applanate, center slightly depressed; surface nearly smooth, fulvous (2A3); margin decurved; context very thin. **Hymenophore** veined, decurrent; folds about 0.1 cm broad, distant, relatively spaced, yellowish (1A2). **Stipe** 2–4 × 0.3–0.8 cm, central, slightly concave and curved in the middle; surface dry, egg-yolk yellow (2A4). Basal mycelium white. **Odor** not distinctive. **Spore print** not obtained.

**Basidiospores** [40/2/1] 8–9–10 × 6.5–7.6–8.5 μm, Q = 1.06–1.36(–1.38), Q_m_ = 1.19 ± 0.09, ellipsoid, rarely subglobose, smooth, slightly thick-walled (up to 0.5 μm), yellowish in KOH. **Basidia** 58–82 × 9–15.5 μm, long, narrow, subcylindrical, slightly thick-walled (up to 0.5 μm), 2–5-spored, yellowish in KOH; sterigmata 3–7 μm in length. **Cystidia** absent. **Pileipellis** a cutis composed of mostly cylindrical, 4–10.5 μm wide, slightly thick-walled (0.5–0.7 μm) hyphae, faintly pale yellow in KOH; terminal cells 45–75 × 5–10 μm, subcylindrical to subclavate with obtuse apex. **Clamp connections** abundant in all parts of the basidioma.

Habitat: Solitary, scattered, or gregarious on the ground of forests dominated by fagaceous trees.

Known distribution: Central China (Hunan Province).

Notes: The collection from central China phylogenetically clustered with one specimen (1D3) identified as *C. tubaeformis* from Japan with strong statistical support (Lineage 11 of Figure 2). Our molecular phylogenetic data also show that specimens identified as *C. tubaeformis* were present in several different parts of the tree (Figure 2). Although the true position of *C. tubaeformis* in the molecular tree should be defined in the future, now we are sure that the Chinese collection in Lineage 11 (Figure 2) is not true *C. tubaeformis*, for the European species has a fuscous or fuacous umber pileus, larger basidiospores measuring 8–11 × 5.5–8 μm, and narrower basidia 60–90 × 8–11 μm (Corner, 1966), which is morphologically different from the Chinese specimen. And thus, the Chinese collection was proposed as a new species.

***Craterellus lutescens*** (Fr.) Fr., Epic. Syst. Mycol. (Upsaliae): 532, 1838 Figures 6A–D, 7.

**FIGURE 6:**
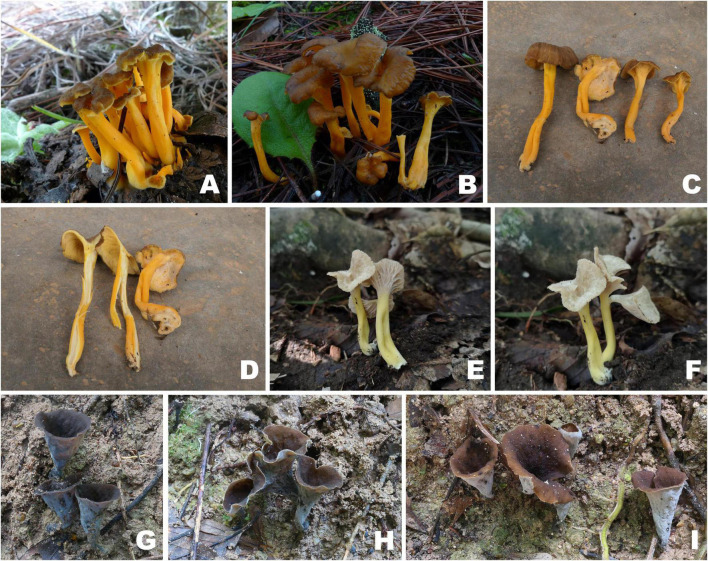
Basidiomata of *Craterellus* species. **(A–D)** *C. lutescens* **(A)** FHMU6547; **(B)** FHMU6548; **(C,D)** FHMU6544; **(E,F)** *C. minor* (FHMU6554, holotype); **(G–I)** *C. parvopullus* **(G)** FHMU6557; **(H)** FHMU6555, holotype; **(I)** FHMU6556. Photos: **(A,B)** L.-P. Tang; **(C,D)** W.-H. Zhang; **(E,F)** P. Zhang; **(G–I)** N.-K. Zeng.

**FIGURE 7:**
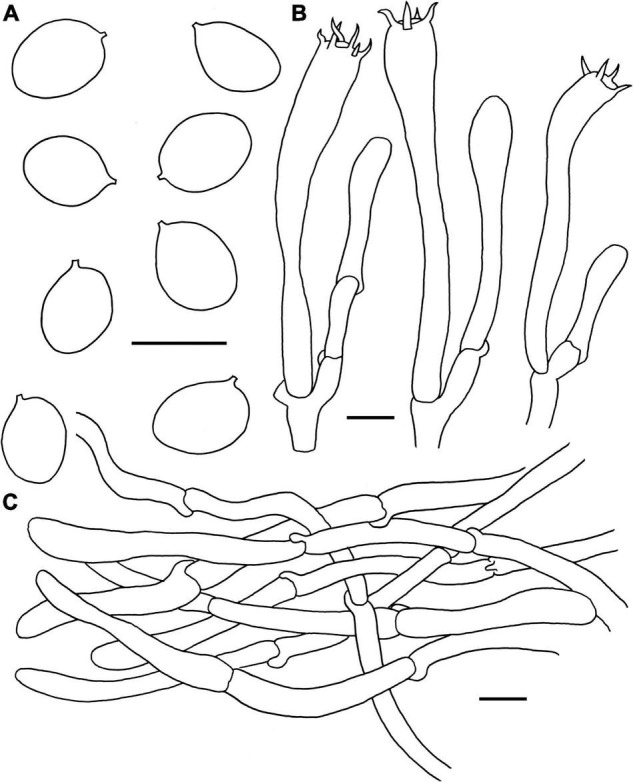
Microscopic features of *Craterellus lutescens* (FHMU6544). **(A)** Basidiospores. **(B)** Basidia. **(C)** Pileipellis. Scale bars = 10 μm. Drawings by Y.-Z. Zhang.

**Basidiomata** very small-sized. **Pileus** about 3 cm diam, nearly convex to applanate, center slightly depressed; margin inrolled; surface nearly smooth, brown (6D5); context about 0.2 cm in thickness, yellowish (2A3). **Hymenophore** veined, sometimes smooth, decurrent; folds very thin, light orange-yellow (4A4) to orange-yellow (4A6). **Stipe** 4–6 × 0.5–0.8 cm, central, cylindrical, hollow; surface dry, sunflower yellow (3A8) to dark yellow (4B8); context yellowish-white (4A2). **Odor** pleasant, milky. **Spore print** not obtained.

**Basidiospores** [240/12/5] (8–)8.5–9.7–11(–11.5) × (6.5–)7–7.8–9(–9.5) μm, Q = 1.13–1.36(–1.46), Q_m_ = 1.23 ± 0.16, ellipsoid, smooth, slightly thick-walled (up to 0.5 μm), pale yellowish in KOH. **Basidia** 61–84 × 7.5–10 μm, long, narrow, subcylindrical, thin to slightly thick-walled (up to 0.5 μm), 4–6-spored, yellowish in KOH; sterigmata 5.5–7 μm in length. **Cystidia** absent. **Pileipellis** a cutis composed of 5.5–10.5 μm wide, slightly thick-walled (0.5–0.7 μm) hyphae, yellowish in KOH; terminal cells 30–58 × 4–8.5 μm, subcylindrical to subclavate with obtuse apex. **Clamp connections** abundant in all parts of the basidioma.

Habitat: Solitary, scattered, or gregarious on the ground of forests dominated by *Pinus yunnanensis* Franch. and *Quercus* L.

Known distribution: Southwestern China (Yunnan Province); Europe (Dahlman et al., 2000).

Specimens examined: CHINA. Yunnan Province: Jianchuan County, Shibaoshan Nature Reserve, near the grotto parking lot, elev. 2,499 m, 16 August 2014, L.P. Tang164*7* (FHMU6547); same location, elev. 2,542 m, 19 August 2014, L.P. Tang1705 (FHMU6548); Lijiang City, bought from a market, 19 August 2020, W.H. Zhang441-1, 441-2, 441-3 (FHMU6544, FHMU6546, and FHMU6545).

Notes: Our collections and three Swedish specimens (UPSF-11789, UPSF-11790, and SS575) of *C. lutescens* phylogenetically group together with strong statistical support (Figure 2). Morphologically, the Chinese specimens match well with those of *C. lutescens* provided by Petersen (1969). Therefore, the specimen from China is recognized as *C. lutescens*.

**FIGURE 8:**
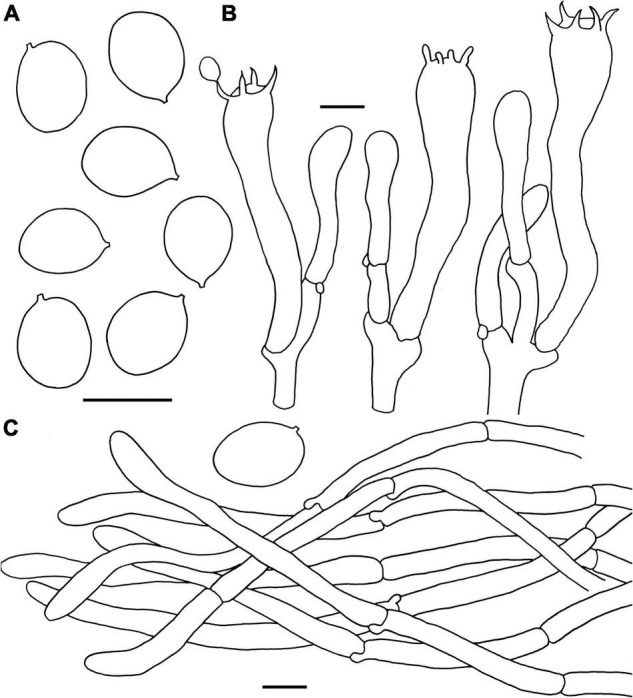
Microscopic features of *Craterellus minor* (FHMU6554, holotype). **(A)** Basidiospores. **(B)** Basidia. **(C)** Pileipellis. Scale bars = 10 μm. Drawings by Y.-Z. Zhang.

***Craterellus minor*** N.K. Zeng, Y.Z. Zhang, P. Zhang & Zhi Q. Liang, sp. nov. Figures 6E,F, 8 MycoBank: MB841974.

Diagnosis: This species is distinguished from others in *Craterellus* by its very small-sized basidioma, a grayish yellow pileus without dark pigments, a veined hymenophore, a lemon-yellow stipe, and the presence of clamp connections in all parts of the basidioma.

Etymology: Latin “*minor*”, refers to very small-sized basidioma of the new species.

Holotype: CHINA. Hunan Province: Sangzhi County, Badagong Mountain, Tianping Mountain, elev. 750 m, 15 September 2020, P. Zhang MHHNU32505 (FHMU6554). GenBank accession number: 28S = OL439684, ITS = OL439553.

**Basidiomata** very small-sized. **Pileus** about 1.7 cm in diam, center strongly depressed; margin inrolled, with irregular small crenulate; surface dry, grayish-yellow (1B2); context very thin, white or whitish (2A1). **Hymenophore** veined, decurrent; folds about 0.1 cm broad, forking gill-folds, white to pale (5A1). **Stipe** 2.6 × 0.3 cm, central, hollow, cylindrical, slightly concave and curved in the middle; surface dry, pale lemon yellow (1A4) with white base (3A1). **Odor** indistinct. **Spore print** not obtained.

**Basidiospores** [40/1/1] (8–)8.5–9.4–10.5 × 7–7.7–8.5 μm, Q = (1.07–)1.12–1.4, Q_m_ = 1.23 ± 0.08, ellipsoid to broadly ellipsoid, smooth, inamyloid, slightly thick-walled (up to 0.5 μm), yellowish in KOH. **Basidia** 56–75 × 8–13 μm, long, narrow, subcylindrical, slightly thick-walled (up to 0.5 μm), 2–5-spored, yellowish in KOH; sterigmata 4.5–8 μm in length. **Cystidia** absent. **Pileipellis** a cutis composed of mostly cylindrical, 5–10 μm wide, slightly thick-walled (up to 0.5 μm) hyphae, faintly pale yellow in KOH; terminal cells 35–85 × 5–7 μm, subcylindrical to subclavate with obtuse apex. **Clamp connections** present in all parts of the basidioma.

Habitat: Solitary to scattered on the ground of forests dominated by fagaceous trees.

Known distribution: Central China (Hunan Province).

Notes: The new collection from central China phylogenetically clustered with one specimen labeled as *C. melanoxeros* (Desm.) Pérez-De-Greg (420526MF0891) also from China with strong statistical support (Figure 2). The Chinese species is morphologically related to European *C. melanoxeros* (SS576). However, *C. melanoxeros* has a large basidioma, a presence of dark pigments, and narrower basidiospores (Dahlman et al., 2000; Akata and Kumbasli, 2014).

**FIGURE 9:**
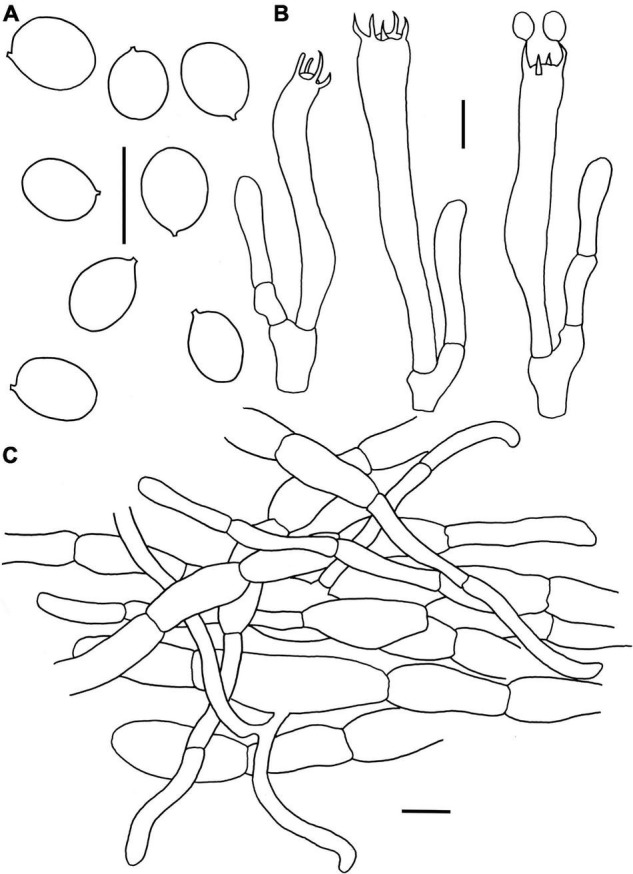
Microscopic features of *Craterellus parvopullus* (FHMU6555, holotype). **(A)** Basidiospores. **(B)** Basidia. **(C)** Pileipellis. Scale bars = 10 μm. Drawings by Y.Z. Zhang.

***Craterellus parvopullus*** N.K. Zeng, Y.Z. Zhang & Zhi Q. Liang, sp. nov. Figures 6G–I, 9 MycoBank: MB841977.

Diagnosis: This species is distinguished from others in *Craterellus* by its basidioma without any obvious demarcation between pileus and stipe, a blackish brown to blackish pileus, a smooth grayish hymenophore, subglobose to ellipsoid or broadly ellipsoid basidiospores, hyphae in pileipellis more or less inflated, but obviously slender in terminations, an absence of clamp connections in all parts of the basidioma, and it is associated with the trees of Dipterocarpaceae.

Etymology: Latin “*parvo*,” meaning small, and “*pullus*,” meaning blackish, refer to the small and blackish pileus of our new species.

Holotype: CHINA. Hainan Province: Wanning County, Bofangling, elev. 80 m, 29 August 2020, N.K. Zeng4913 (FHMU6555). GenBank accession number: 28S = OL439685, ITS = OM334829.

**Basidiomata** very small to small-sized. **Pileus** 1.8–4.6 cm diam, infundibuliform; margin slightly incurved, wavy, irregularly folded; surface dry, blackish brown (6F7) to black (5F1); context very thin, grayish (1E1). **Hymenophore** smooth to slightly folded, ashen gray (4B1). **Stipe** 1.2–2.6 × 0.15–0.4 cm, confluent with pileus, hollow; surface dry, ashen gray (4B1); context very thin, grayish (1E1). **Odor** not distinctive. **Spore print** not obtained.

**Basidiospores** [80/16/3] (6.5–)7–7.7–8.5(–9) × (5–)5.5–6.2–7(–7.5) μm, Q = (1.07–)1.14–1.42(–1.45), Q_m_ = 1.25 ± 0.09, subglobose to ellipsoid or broadly ellipsoid, smooth, slightly thick-walled (up to 0.5 μm), yellowish in KOH. **Basidia** 53–73 × 7–10 μm, subcylindrical to subclavate, slightly thick-walled (up to 0.5 μm), 3–5-spored, hyaline or yellowish in KOH; sterigmata 4–6.5 μm in length. **Cystidia** absent. **Pileipellis** a cutis composed of mostly cylindrical, occasionally branched hyphae, hyphae 8–14 μm wide, but slender in terminations (3–6 μm wide), thin- to thick-walled (up to 1.5 μm), yellowish in KOH; terminal cells 21–46 × 3–9 μm, clavate or subcylindrical with obtuse apex. **Clamp connections** absent in all tissues.

Habitat: Gregarious on the ground in forests of *Vatica mangachapoi* Blanco.

Known distribution: Southern China (Hainan Province).

Additional specimens examined: CHINA. Hainan Province: Wanning County, Bofangling, elev. 80 m, 29 August 2020, N.K. Zeng4911, 4912 (FHMU6557, FHMU6556).

Notes: The Chinese *C. atrobrunneolus* T. Cao & H.S. Yuan, *C. badiogriseus* T. Cao & H.S. Yuan, *C. croceialbus* T. Cao & H.S. Yuan, *C. macrosporus* T. Cao & H.S. Yuan, and *C. squamatus* T. Cao & H.S. Yuan are morphologically similar to *C. parvopullus*. However, *C. atrobrunneolus* is distributed in subtropical areas (Cao et al., 2021a), while *C. badiogriseus*, *C. croceialbus*, *C. macrosporus*, and *C. squamatus* grow in temperate regions (Cao et al., 2021b); all of them are not associated with trees of Dipterocarpaceae (Cao et al., 2021a,b). Moreover, *C. atrobrunneolus* has smaller basidiospores measuring (6.2–)6.5–7.8(–8) × (4.2–)4.5–6(–6.2) μm (Cao et al., 2021a); *C. badiogriseus* has larger basidiospores measuring (7.5–)8–10.5(–11) × (6.5–)6.8–7.5(–8) μm, and a pileipellis composed of thick-walled hyphae without slender terminations (Cao et al., 2021b); *C. croceialbus* has a brown pileus with an orange-white margin, larger basidiospores measuring (9–)10–12(–12.5) × (6.5–)6.8–8(–8.2) μm, and a pileipellis composed of hyphae without slender terminations (Cao et al., 2021b); *C. macrosporus* has a brown pileus, larger basidiospores measuring (12.5–)12.8–14.5(–15) × (8.8–)9–11(–11.5) μm, and a pileipellis composed of thin-walled hyphae without slender terminations (Cao et al., 2021b); *C. squamatus* has a squamulose pileus, larger basidiospores measuring (11.5–)12–13.8(–14) × (8.2–)8.5–9.5(–10) μm, and a pileipellis composed of thick-walled hyphae without slender terminations (Cao et al., 2021b).

Besides the five species found in China, Malaysian *C. cornucopioides* var. *mediosporus* Corner and *C. verrucosus* Massee, European *C. cornucopioides*, North American *C. atrocinereus* D. Arora & J.L. Frank, *C. calicornucopioides* D. Arora & J.L. Frank and *C. fallax* A.H. Sm are also morphologically similar to *C. parvopullus.* However, *C. verrucosus* has a rugulose hymenophore, larger basidiospores measuring 8–10 × 6.5–8 μm, and wider hyphae (up to 20 μm) more or less vertically arranged in the pileipellis (Corner, 1966); *C. cornucopioides* var. *mediosporus* has larger basidiospores measuring 8–10 × 6.5–7.5 μm, and a pileipellis composed of uninflated hyphae (Corner, 1966); *C. cornucopioides* s.s. has larger basidiospores measuring (7–)11–15(–20) × (5–)7(–11) μm, and its distribution in temperate areas (Pilz et al., 2003); *C. atrocinereus* has larger basidiospores measuring 8–10 × 4.5–6 μm, a prominently folded, distinctly thick hymenium, and groups on the ground under hardwoods, especially *Quercus* and *Neolithocarpus* (Frank, 2015); *C. calicornucopioides* has larger basidiospores measuring 11–14 × 8–10 μm, a presence of abundant clamp connections, and is mainly distributed with *Quercus*, *Arctostaphylos*, *Vaccinium* and *Arbutus* (Frank, 2015); *C. fallax* has larger basidiospores measuring 10–13 × 7–9 μm, and is mainly distributed in a broad host range, including Pinaceae (*Pinus* and *Tsuga*) and Fagaceae (*Quercus* and *Castanea*) (Matheny et al., 2010). Phylogenetically, *C. parvopullus* is not closely related to *C. atrobrunneolus*, *C. atrocinereus*, *C. calicornucopioides*, *C. cornucopioides*, and *C. fallax* (Figure 2).

### Key to Known *Craterellus* Species in China

1. Without any obvious demarcation between pileus and stipe……………………………………………………………………………….. 2
1. Obvious demarcation between pileus and stipe……………….8
2. Pileus vivid yellow to orange…………………………………*C. aureus*
2. Pileus brown, gray brown, dark brown to almost black……3
3. Pileal surface scabrous…………………………………….*C. squamatus*
3. Pileal surface subglabrous to glabrous……………………………..4
4. Pileal surface blackish brown, blackish to almost black……5
4. Pileal surface brown, gray-brown to dark brown, without black tinge………………………………………………………………………7
5. Hyphal width in pileipellis usually uneven, obviously slender in terminations, and distributed in tropical areas………………………………………………………………*C. parvopullus*
5. Hyphal width in pileipellis usually even, and distributed in subtropical or temperate areas………………………………………..6
6. Basidiospores larger [(7.5–)8–10.5(–11) × (6.5–)6.8–7.5(–8) μm]………………………………………………………….*C. badiogriseus*
6. Basidiospores smaller [(6.2–)6.5–7.8(–8) × (4.2–)4.5–6(–6.2) μm]…………………………………………………..*C. atrobrunneolus*
7. Pileal margin orange-white, basidiospores smaller [(9–)10–12(–12.5) × (6.5–)6.8–8(–8.2) μm]……………..*C. croceialbus*
7. Pileal margin dark brown, basidiospores larger [(12.5–)12.8–14.5(–15) × (8.8–)9–11.0(–11.5) μm].*C. macrosporus*
8. Basidomata very pale, whitish, hyphal clamp connections absent, grow on dead wood…………………………………..*C. albidus*
8. Basidiomata brown, yellow, hyphal clamp connections abundant, grow on ground……………………………………………..9
9. Pileus brown, hymenophore veined, sometimes smooth……………………………………………………………….*C. lutescens*
9. Pileus fulvous, grayish-yellow, hymenophore veined, never smoot……………………………………………………………………………10
10. Stipe egg-yolk yellow………………………………………….*C. fulviceps*
10. Stipe pale lemon yellow………………………………………….*C. minor*

## Discussion

### *Craterellus cornucopioides* and *Craterellus tubaeformis* Complexes

*Craterellus cornucopioides*, originally described in Europe, was previously considered a widely distributed species (Akata and Kumbasli, 2014). However, recent studies have indicated that *C. cornucopioides* represents a species complex rather than a single widespread species (Dahlman et al., 2000). Our molecular phylogenetic data also show that specimens identified as *C. cornucopioides* were present in several different parts of the tree (Figure 2). Interestingly, collections of *C. cornucopioides* from Europe were present in more than one part of the tree (Figure 2). The species concept of *C. cornucopioides* should be confirmed by obtaining collections and DNA sequences from the holotype locality. *Craterellus cornucopioides* s. str. likely occurs in fewer areas of Europe; one specimen identified as *C. cornucopioides* from Tibet, western China (Lineage 8 in Figure 2), might represent another species. *Craterellus tubaeformis* was also present in several parts of the tree (Figure 2), which indicates that *C. tubaeformis* represents a species complex rather than a single widespread species; the collections identified as *C. tubaeformis* in China from previous studies should be re-evaluated.

### Species Diversity of *Craterellus* in China

High species diversity of *Craterellus* in China was revealed in this study, with fourteen species-level lineages identified (Figure 2). Three lineages (3, 11, and 12) were described as new species, viz. *C. minor*, *C. parvopullus*, and *C. fulviceps.* Eight lineages (1, 4–7, 9, 10, and 14) represent previously described species, viz. *C. albidus*, *C. atrobrunneolus*, *C. aureus*, *C. badiogriseus*, *C. croceialbus*, *C. lutescens*, *C. macrosporus*, and *C. squamatus.* Three lineages (2, 8, and 13) remain undescribed because of insufficient materials. Five additional species have been reported from China, viz. *C. cornucopioides*, *C. cornucopioides* var. *parvisporus*, *C. luteus*, *C. odoratus*, and *C. tubaeformis*. *Craterellus luteus* is a synonym of *C. aureus*, and the occurrence of *C. cornucopioides*, *C. cornucopioides* var. *parvisporus*, *C. odoratus*, and *C. tubaeformis* has not yet been confirmed in China.

### Phylogenetic Relationships and Geographic Divergence of *Craterellus*

Our molecular phylogenetic data based on two-locus DNA sequences (28S + ITS) with a large number of collections from China have uncovered useful information regarding the phylogeny and geography of *Craterellus*. Our data indicate that the affinities of *Craterellus* species between China and Europe, North America, and Australia are evident (Figure 2); for example, *C. lutescens* (Lineage 14 in Figure 2) is found in China, Europe, and North America; *C. badiogriseus* (Lineage 9 in Figure 2) is associated with one specimen (LMAC6b-09) from Europe; *C. aureus* (Lineage 1 in Figure 2), and two Chinese specimens (FHMU6551 and FHMU6552) (Lineage 2 in Figure 2) of *Craterellus* are closely related to North American *C. odoratus*; *C. parvopullus* (Lineage 3 in Figure 2) is closely related to two specimens (GMB-2014 MEL:2382717 and GMB-2014 MEL:2383015) from Australia; *C. macrosporus* (Lineage 6 in Figure 2), *C. squamatus* (Lineage 5 in Figure 2), and two North American specimens (NC-8338 and FLAS-F-60401) labeled as *C.* sp. and *C. fallax*, respectively, are in the same clade; a Chinese specimen (ECM90) labeled as *C.* sp. (Lineage 13 in Figure 2) is closely related to one collection (M66A9) from Mexico. Moreover, *C. fulviceps* (lineage 11 in Figure 2) is found in China and Japan; *C. parvopullus* (lineage 3 in Figure 2) is associated with two specimens (LAM 0254 and AWW263) from Malaysia.

We also noted that there is little or no statistical support in some deeper nodes of the phylogeny, although the molecular data provided new insights into the phylogeny and geography of *Craterellus* with a large number of collections from China included. In the future, with more genes investigated and more *Craterellus* species discovered, a molecular phylogenetic tree of *Craterellus* should be constructed on the basis of the present data, which will provide more interesting information.

## Disclosure

All the experiments undertaken in this study comply with the current laws of the People’s Republic of China.

## Data Availability Statement

The datasets presented in this study can be found in online repositories. The names of the repository/repositories and accession number(s) can be found below: National Center for Biotechnology Information (NCBI) GenBank, https://www.ncbi.nlm.nih.gov/genbank/, OL439672–OL439687, OM334827–OM334829, OL439545–OL439553, OM469019–OM469020 and MycoBank, https://www.mycobank.org/, MB841969, MB841974, MB841977.

## Author Contributions

Z-QL and N-KZ: conceptualization and writing—original draft preparation. Y-ZZ: methodology, performing the experiment, and formal analysis. N-KZ, PZ, L-PT, Z-HC, M-SS, Y-JH, H-YH, and W-HZ: resources. N-KZ, BB, Z-QL, PZ, H-YH, and W-HZ: writing—review and editing. N-KZ and Z-QL: supervision. N-KZ: project administration and funding acquisition. All authors contributed to the article and approved the submitted version.

## Conflict of Interest

The authors declare that the research was conducted in the absence of any commercial or financial relationships that could be construed as a potential conflict of interest. The handling editor BD declared a past co-authorship with the author BB.

## Publisher’s Note

All claims expressed in this article are solely those of the authors and do not necessarily represent those of their affiliated organizations, or those of the publisher, the editors and the reviewers. Any product that may be evaluated in this article, or claim that may be made by its manufacturer, is not guaranteed or endorsed by the publisher.

## Footnotes

1
https://www.ncbi.nlm.nih.gov/genbank

## References

[B1] Akata I. Kumbasli M. (2014). A new and rare record for turkish *Cantharellus*. *Biodivers. Conserv.* 7 143–145.

[B2] An D. Y. Liang Z. Q. Jiang S. Su M. S. Zeng N. K. (2017). *Cantharellus hainanensis*, a new species with a smooth hymenophore from tropical China. *Mycoscience* 58 438–444. 10.1016/j.myc.2017.06.004

[B3] Arraiano-Castilho R. Bidartondo M. I. Niskanen T. Clarkson J. J. Brunner I. Zimmermann S. (2020). Habitat specialization controls ectomycorrhizal fungi above the treeline in the European Alps. *New Phytol.* 229 2901–2916. 10.1111/nph.17033 33107606

[B4] Bas C. (1969). Morphology and subdivision of *Amanita* and a monograph of its section *Lepidella*. *Persoonia* 5 285–579.

[B5] Beluhan S. Ranogajec A. (2011). Chemical composition and non-volatile components of Croatian wild edible mushrooms. *Food Chem.* 124 1076–1082. 10.1016/j.foodchem.2010.07.081

[B6] Berkeley M. J. Curtis M. A. (1860). Characters of new fungi, collected in the north pacific exploring expedition by Charles Wright. *Proc. Am. Acad. Arts. Sci.* 4 111–130.

[B7] Bijeesh C. Kumar A. M. Vrinda K. B. Pradeep C. K. (2018). Two new species of *Craterellus* (Cantharellaceae) from tropical India. *Phytotaxa* 346 157–168. 10.11646/phytotaxa.372.1.5

[B8] Buyck B. Kauff F. Eyssartier G. Couloux A. Hofstetter V. (2014). A multilocus phylogeny for worldwide *Cantharellus* (Cantharellales, Agaricomycetidae). *Fungal Divers.* 64 101–121. 10.1007/s13225-013-0272-3

[B9] Cao T. Yu J. R. Hu Y. P. Yuan H. S. (2021a). *Craterellus atrobrunneolus* sp. nov. from southwestern China. *Mycotaxon* 136 59–71. 10.5248/136.59

[B10] Cao T. Hu Y. P. Yu J. R. Wei T. Z. Yuan H. S. (2021b). A phylogenetic overview of the *Hydnaceae* (Cantharellales, Basidiomycota) with new taxa from China. *Stud. Mycol.* 99 100–121. 10.1016/j.simyco.2021.100121 35035603 PMC8717575

[B11] Carriconde F. Gardes M. Bellanger J. M. Letellier K. Gigante S. Gourmelon V. (2019). Host effects in high ectomycorrhizal diversity tropical rainforests on ultramafic soils in New Caledonia. *Fungal Ecol.* 39 201–212. 10.1016/j.funeco.2019.02.006

[B12] Castresana J. (2000). Selection of conserved blocks from multiple alignments for their use in phylogenetic analysis. *Mol. Biol. Evol.* 17 540–552. 10.1093/oxfordjournals.molbev.a026334 10742046

[B13] Corner E. J. H. (1966). *A Monograph of Cantharelloid Fungi.* London, EN: Oxford University Press.

[B14] Dahlman M. Danell E. Spatafora J. W. (2000). Molecular systematics of *Craterellus*: cladistic analysis of nuclear LSU rDNA sequence data. *Mycol. Res.* 104 388–394. 10.1017/S0953756299001380

[B15] Das K. Ghosh A. Chakraborty D. Li J. Qiu L. Baghela A. (2017). Fungal biodiversity profiles 31–40. *Mycologia* 38 353–406. 10.7872/crym/v38.iss3.2017.353

[B16] Disyatat N. R. Yomyart S. Sihanonth P. Piapukiew J. (2016). Community structure and dynamics of ectomycorrhizal fungi in a dipterocarp forest fragment and plantation in Thailand. *Plant Ecol. Divers.* 9 577–588. 10.1080/17550874.2016.1264018

[B17] Edgar R. C. (2004). MUSCLE: multiple sequence alignment with high accuracy and high throughput. *Nucleic Acids Res.* 32 1792–1797. 10.1093/nar/gkh340 15034147 PMC390337

[B18] Edwards I. P. Cripliver J. L. Gillespie A. R. Johnsen K. H. Scholler M. Turco R. F. (2004). Nitrogen availability alters macrofungal basidiomycete community structure in optimally fertilized loblolly pine forests. *New Phytol.* 162 755–770. 10.1111/j.1469-8137.2004.01074.x 33873755

[B19] Fan X. D. Chen Y. H. Chang J. Chen J. (2014). Structure and antitumor activity of water-soluble polysaccharide from *Craterellus cornucopioides*. *Mod. Food Sci. Technol.* 30 50–54+79. 10.1016/j.carbpol.2018.02.077 29628263

[B20] Federico D. R. Manuel A. Francesco T. (2020). The history of conifers in central Italy supports long-term persistence and adaptation of mesophilous conifer fungi in arbutus-dominated shrublands. *Rev. Palaeobot. Palynol.* 282 104300–104314. 10.1016/j.revpalbo.2020.104300

[B21] Feibelman T. P. Doudrick R. L. Cibula W. G. Bennett J. W. (1997). Phylogenetic relationships within the Cantharellaceae inferred from sequence analysis of the nuclear large subunit rDNA. *Mycol. Res.* 101 1423–1430. 10.1017/S0953756297004115

[B22] Frank J. L. (2015). *Craterellus atrocinereus D. Arora & J.L. Frank*. *Index Fungorum* 249:1.

[B23] Hall T. A. (1999). BioEdit: a user-friendly biological sequence alignment editor and analyses program for Windows 95/98/NT. *Nucleic Acids Symp. Ser.* 41 95–98.

[B24] Harrington T. J. Mitchell D. T. (2002). Characterization of *Dryas octopetala* ectomycorrhizas from limestone karst vegetation, western Ireland. *Can. J. Bot.* 80 970–982. 10.1139/b02-082

[B25] Hembrom M. E. Das K. Adhikari S. Parihar A. Buyck B. (2017). First report of Pterygellus from Rajmahal hills of Jharkhand (India) and its relation to *Craterellus* (Hydnaceae, Cantharellales). *Phytotaxa* 306 201–210. 10.11646/phytotaxa.306.3.2

[B26] Hibbett D. S. Bauer R. Binder M. Giachini A. J. Hosaka K. Justo A. (2014). “Agaricomycetes,” in *The Mycota vol.VII, Systematics and Evolution part A*, 2nd Edn, eds McLaughlin D. J. Spatafora J. W. (Berlin, DE: SpringerVerlag), 373–429. 10.1007/978-3-642-55318-9_14

[B27] Huang Y. Zhang S. B. Chen H. P. Zhao Z. Z. Li Z. H. Feng T. (2016). New acetylenic acids and derivatives from the Basidiomycete *Craterellus lutescens* (Cantharellaceae). *Fitoterapia* 115 177–181. 10.1016/j.fitote.2016.10.006 27773765

[B28] Huang Y. Zhang S. B. Chen H. P. Zhao Z. Z. Zhou Z. Y. Li Z. H. (2017). New acetylenic acids and derivatives from the edible mushroom *Craterellus lutescens* (Cantharellaceae). *J. Agric. Food Chem.* 65 3835–3841. 10.1021/acs.jafc.7b00899 28468498

[B29] Huelsenbeck J. P. Ronquist F. (2005). “Bayesian analysis of molecular evolution using MrBayes,” in *Statistical Methods in Molecular Evolution*, ed. Nielsen R. (New York, NY: Springer), 183–226. 10.1007/0-387-27733-1_7

[B30] James T. Y. Kauff F. Schoch C. Matheny P. B. Hofstetter V. Cox C. (2006). Reconstructing the early evolution of the fungi using a six gene phylogeny. *Nature* 443 818–822. 10.1038/nature05110 17051209

[B31] Knopf A. A. (1981). *The Audubon Society field guide to North American Mushrooms.* New York, NY: Lincoff.

[B32] Ko P. Y. Lee S. H. Kim T. H. Hong K. S. Choe S. Y. Jeun Y. C. (2020). Distribution of spontaneously growing mushrooms in the Wolchulsan National Park. *J. Mushrooms* 18 201–207. 10.14480/JM.2020.18.3.201

[B33] Kornerup A. Wanscher J. H. (1981). *Taschenlexikon der Farben 3.* Northeim, DE: Muster-Schmidt Verlagsgesellschaft.

[B34] Kotowski M. A. Pietras M. Łuczaj Ł (2019). Extreme levels of mycophilia documented in Mazovia, a region of Poland. *J. Ethnobiol. Ethnomed.* 15 12–31. 10.1186/s13002-019-0291-6 30755235 PMC6371552

[B35] Kumari D. Upadhyay R. C. Reddy M. S. (2012). *Craterellus indicus* sp. nov., a new species associated with *Cedrus deodara* from the western Himalayas, India. *Mycol. Prog.* 11 769–774. 10.1007/s11557-011-0788-4

[B36] Li R. C. (1996). Resources of *Cantharellus* in Yunnan. *Edible Fungi China* 15 18–20.

[B37] Li T. H. Chen Y. Q. Qu L. H. Lu Y. J. Song B. (1999). Partial 25S rDNA sequence of *Cantharellus* and ITS phylogenetic implications. *Mycosystema* 18 12–19.

[B38] Li X. (2005). Common large-scale edible fungi in Simao, Yunnan. *J. Simao Teach. Coll.* 3 25–28.

[B39] Liu Y. T. Sun J. Luo Z. Y. Rao S. Q. Su Y. J. Xu R. R. (2012). Chemical composition of five wild edible mushrooms collected from Southwest China and their antihyperglycemic and antioxidant activity. *Food Chem. Toxicol.* 50 1238–1244. 10.1016/j.fct.2012.01.023 22300772

[B40] Matheny P. B. Austin E. A. Birkebak J. M. Wolfenbarger A. D. (2010). *Craterellus fallax*, a black trumpet mushroom from eastern North America with a broad host range. *Mycorrhiza* 20 569–575. 10.1007/s00572-010-0326-2 20602121

[B41] Matheny P. B. Wang Z. Binder M. Curtis J. M. Lim Y. W. Nilsson R. H. (2007). Contributions of rpb2 and *tef1* to the phylogeny of mushrooms and allies (Basidiomycota, Fungi). *Mol. Phylogenet. Evol.* 43 430–51. 10.1016/j.ympev.2006.08.024 17081773

[B42] Mešić A. Šamec D. Jadan M. Bahun V. Tkalčec Z. (2020). Integrated morphological with molecular identification and bioactive compounds of 23 Croatian wild mushrooms samples. *Food Biosci.* 37 100720–100730. 10.1016/j.fbio.2020.100720

[B43] Miller M. A. Pfeiffer W. Schwartz T. (2011). “The CIPRES science gateway: a community resource for phylogenetic analyses,” in *Proceedings of the 2011 TeraGrid Conference: extreme Digital Discovery*, (New York, NY: ACM), 41.

[B44] Miyamoto Y. Nakano T. Hattori M. Nara K. (2014). The mid-domain effect in ectomycorrhizal fungi: range overlap along an elevation gradient on Mount Fuji. *Japan. ISME J.* 8 1739–1746. 10.1038/ismej.2014.34 24621523 PMC4817612

[B45] Miyamoto Y. Narimatsu M. Nara K. (2018). Effects of climate, distance, and a geographic barrier on ectomycorrhizal fungal communities in Japan: a comparison across Blakiston’s Line. *Fungal Ecol.* 33 125–133. 10.1016/j.funeco.2018.01.007

[B46] Morris M. H. Perez-Perez M. A. Smith M. E. Bledsoe C. S. (2008). Multiple species of ectomycorrhizal fungi are frequently detected on individual oak root tips in a tropical cloud forest. *Mycorrhiza* 18 375–383. 10.1007/s00572-008-0186-1 18704515

[B47] Naseer A. Khalid A. N. (2018). A new record of genus *Craterellus*, edible basidiomycotous fungus from Pakistan. *Saudi J. Med. Pharm. Sci*. 4 656–659. 10.21276/sjmps.2018.4.6.2

[B48] Nylander J. A. A. (2004). *MrModeltest 2.3. Program Distributed by the Author.* Uppsala: Uppsala University.

[B49] O’Callaghan Y. C. O’Brien N. M. Kenny O. Harrington T. Brunton N. Smyth T. J. (2014). Anti-inflammatory effects of wild Irish mushroom extracts in RAW264.7 mouse macrophage cells. *J. Med. Food* 18 202–7. 10.1089/jmf.2014.0012 25136763

[B50] Petersen R. H. (1969). Notes on cantharelloid fungi—II. Some new taxa, and notes on Pseudocraterellus. *Persoonia* 5 211–223.

[B51] Petersen R. H. (1979a). Notes on cantharelloid fungi. IX. Illustrations of new or poorly understood taxa. *Nova Hedwigia* 31 1–23.

[B52] Petersen R. H. (1979b). Notes on cantharelloid fungi. X. *Cantharellus confluens* and *C. lateritius*, *Craterellus odoratus* and *C. aureus*. *Sydowia* 32 198–208.

[B53] Pilz D. Norvell L. Danell E. Molina R. (2003). *Ecology and management of commercially harvested chanterelle mushrooms.* Washington, DC: US Department of Agriculture, Forest Service.

[B54] Porter T. M. Skillman J. E. Moncalvo J. M. (2008). Fruiting body and soil rDNA sampling detects complementary assemblage of Agaricomycotina (Basidiomycota, Fungi) in a hemlock-dominated forest plot in southern Ontario. *Mol. Ecol.* 17 3037–3050. 10.1111/j.1365-294X.2008.03813.x 18494767

[B55] Raja H. A. Baker T. R. Little J. G. Oberlies N. H. (2017). DNA barcoding for identification of consumer-relevant mushrooms: a partial solution for product certification? *Food Chem.* 214 383–392. 10.1016/j.foodchem.2016.07.052 27507489

[B56] Shao S. C. Buyck B. Hofstetter V. Tian Y. H. Yu F. Q. Liu P. G. (2014). *Cantharellus hygrophorus*, a new species in subgenus *Afrocantharellus* from tropical southwestern China. *Cryptogamie Mycol.* 35 283–291. 10.7872/crym.v35.iss3.2014.283

[B57] Smith S. A. Dunn C. W. (2008). Phyutility: a phyloinformatics tool for trees, alignments andmolecular data. *Bioinformatics* 24 715–716. 10.1093/bioinformatics/btm619 18227120

[B58] Stamatakis A. (2006). RAxML-VI-HPC: maximum likelihoodbased phylogenetic analyses with thousands of taxa and mixed models. *Bioinformatics* 22 2688–2690. 10.1093/bioinformatics/btl446 16928733

[B59] Tibuhwa D. D. Savić S. Tibell L. Kivaisi A. K. (2012). *Afrocantharellus* gen. stat. nov. is part of a rich diversity of African Cantharellaceae. *IMA Fungus* 3 25–38. 10.5598/imafungus.2012.03.01.04 23155498 PMC3399100

[B60] Urban A. Puschenreiter M. Strauss J. Gorfer M. (2008). Diversity and structure of ectomycorrhizal and co-associated fungal communities in a serpentine soil. *Mycorrhiza* 18 339–354. 10.1007/s00572-008-0189-y 18677625

[B61] Vilgalys R. Hester M. (1990). Rapid genetic identification and mapping of enzymatically amplified ribosomal DNA from several *Cryptococcus* species. *J. Bacteriol.* 172 4238–4246. 10.1128/jb.172.8.4238-4246.1990 2376561 PMC213247

[B62] Wang X. H. Liu P. G. Yu F. Q. (2004). *Color atlas of wild commercial mushrooms in Yunnan.* Kunming: Yunnan Science and Technology Press.

[B63] White T. J. Bruns T. Lee S. Taylor J. W. (1990). “Amplification and direct sequencing of fungal ribosomal RNA genes for phylogenies,” in *PCR Protocols: A Guide to Methods and Applications*, eds Innis M. A. Gelfand D. H. Sninsky J. J. White T. J. (New York, NY: Academic Press), 315–322. 10.1016/B978-0-12-372180-8.50042-1

[B64] Wilson A. W. Aime M. C. Dierks J. Mueller G. M. Henkel T. W. (2012). Cantharellaceae of Guyana I: new species, combinations and distribution records of *Craterellus* and a synopsis of known taxa. *Mycologia* 104 1466–1477. 10.3852/11-412 22684285

[B65] Xiao Z. D. Song B. Li T. H. Deng C. Y. Huang H. (2012). Resource of Cantharellaceae from Chebaling Nature Reserve, Guangdong Province. *Edible Fungi China* 31 12–13.

[B66] Zhang J. Wu D. I. Deng C. Y. Zhang M. Dauner L. Wijayawardene N. N. (2020). A new species of *Craterellus* (Cantharellales, Hydnaceae) from Guizhou Province, China. *Phytotaxa* 472 259–268. 10.11646/phytotaxa.472.3.4

[B67] Zhang L. Shen Y. Wang F. Leng Y. Liu J. K. (2010). Rare merosesquiterpenoids from basidiomycete *Craterellus odoratus* and their inhibition of 11β-hydroxysteroid dehydrogenases. *Phytochemistry* 71 100–103. 10.1016/j.phytochem.2009.09.020 19879607

[B68] Zhang Y. Mo M. Z. Yang L. Mi F. Cao Y. Liu C. L. (2021). Exploring the species diversity of edible mushrooms in Yunnan, southwestern China, by DNA barcoding. *J. Fungi* 7 310–333. 10.3390/jof7040310 33920593 PMC8074183

[B69] Zhong X. R. Li T. H. Jiang Z. D. Deng W. Q. Huang H. (2018). A new yellow species of *Craterellus* (Cantharellales, Hydnaceae) from China. *Phytotaxa* 360 35–44. 10.11646/phytotaxa.360.1.3

[B70] Zhou G. Y. Guo L. Li L. Li H. (2011). rDNA internal transcribed spacer sequence analysis of *Craterellus tubaeformis* from north America and Europe. *Can. J. Microbiol.* 57 29–32. 10.1139/W10-098 21217794

